# Optimal degrees of freedom of the lower extremities for human walking and running

**DOI:** 10.1038/s41598-023-43239-y

**Published:** 2023-09-27

**Authors:** Shoma Kudo, Masahiro Fujimoto, Takahiko Sato, Akinori Nagano

**Affiliations:** 1 National Institute of Advanced Industrial Science and Technology (AIST), Kashiwa II Campus, University of Tokyo, 6-2-3 Kashiwanoha, Kashiwa, Chiba Japan; 2 Biwako Professional University of Rehabilitation, Higashiomi, Shiga, Japan; 3 College of Sport and Health Science, Ritsumeikan University, Kusatsu, Shiga, Japan

**Keywords:** Anatomy, Musculoskeletal system, Biomedical engineering

## Abstract

Determining the degrees of freedom (DOF) of the linked rigid-body model, representing a multi-body motion of the human lower extremity, is one of the most important procedures in locomotion analysis. However, a trade-off exists between the quality of data fitting and the generalizability of the model. This study aimed to determine the optimal DOF of the model for the lower extremities that balance the goodness-of-fit and generalizability of the model during walking and running using Akaike’s information criterion (AIC). Empirically obtained kinematic data for the lower extremities during walking and running were fitted by models with 9, 18, or 22 DOF. The relative quality of these models was assessed using their bias-corrected AIC (cAIC) value. A significant simple main effect of the model was found on the cAIC value for both walking and running conditions. Pairwise comparisons revealed that the cAIC value of the 18-DOF model was significantly smaller than that of the 9-DOF (walking: *p* < 0.001, running: *p* = 0.010) and 22-DOF (walking: *p* < 0.001, running: *p* < 0.001) models. These findings suggest that the 18-DOF model is optimal for representing the lower extremities during walking and running, in terms of goodness-of-fit and generalizability.

## Introduction

Determining the degrees of freedom (DOF) for a joint of a linked rigid-body model of the human lower extremity is one of the most important procedures in human locomotion analysis. The number of DOFs is a crucial factor in determining how rigid bodies are permitted to move relative to each other. It affects the validity of kinematic data of humans during locomotion quantified when utilizing the inverse and forward dynamics analyses. However, the number of DOFs best suited for representing the motion of the lower limb during locomotion remains unclear, and models of the lower extremity with different numbers of DOFs have been used to analyze locomotion^1–4^.

When determining the DOFs of a rigid-body model, a trade-off exists between the goodness-of-fit of the model and its generalizability^5^. A model with a larger DOF can better represent the lower extremities than a model with a smaller DOF, in terms of the quality of fitting to the data. However, the more DOFs the model has, the more complicated the model becomes, impairing its ability to be generalized because it is too specific for a particular dataset of participants. Therefore, this trade-off must be considered when determining the optimal model number of DOF for the lower extremities that balances the model’s goodness of fit and generalizability.

Akaike’s information criterion (AIC), which addresses this trade-off, can be used to select the optimal model complexity. The AIC is a composite measure comprising the sum of two terms: the maximized value of the likelihood function for the estimated model and a function of the DOF that declines with additional parameters, thus penalizing increasing model complexity^6^. Hence, the AIC rewards goodness-of-fit and includes a penalty as a function of the number of estimated parameters. Therefore, the AIC can be used for optimal model selection.

This study, therefore, aimed to determine the optimal number of DOF of the model for the lower extremities to balance the goodness-of-fit and generalizability of the model during human locomotion. The lower extremities were modeled with different numbers of DOF, and the relative quality was assessed using the AIC and compared among the models during walking and running for optimal model selection.

## Methods

### Participants

Ten adult males (mean ± standard deviation (SD) age: 22.6 ± 1.5 years, height: 1.70 ± 0.05 m, body mass: 64.6 ± 6.0 kg) participated in this study. All participants reviewed and signed an informed consent form. All participants were asked to review and sign an informed consent form prior to participating in the study. The study protocol was conducted in accordance with the guidelines proposed in the Declaration of Helsinki and was reviewed and approved by the Institutional Review Board at Ritsumeikan University, Biwako-Kusatsu Campus in Japan.

### Data collection

The participants were instructed to walk continuously and run on a 20 m circuit runway five times while maintaining their preferred speed. Three-dimensional position data of the lower extremities along a straight distance of 5 m of the runway (from a 2.5–7.5 m section of the runway) during the third lap in each trial were recorded using a 24-camera-motion capture system at 250 Hz (MAC3D, Motion Analysis Corporation, California, USA). A total of 26 reflective markers were placed on each participant’s body at the anatomical landmarks to measure the three-dimensional positions of the segments^7^.

### Data analysis

Three linked rigid body models with different DOF represented the lower extremities in each trial. These models have 9 (9-DOF model), 18 (18-DOF model), and 22 DOFs (22-DOF model). The 9-DOF model is planar in the sagittal plane and has often been used as a simple model in human locomotion analysis^3,8,9^. The 18-DOF model has been widely used in the three-dimensional musculoskeletal computer simulations of human locomotion^1,2^. The 22-DOF model was constructed as the most complex model under the experimental conditions in this study. The DOFs of each model are listed in Table 1.

**Table 1:** Joints and their degrees of freedom implemented in the models.

Segment	Joint	Movements	DOF		
			9-DoF	18-DoF	22-DoF
Pelvis	GCS	x (Anterior–posterior direction)	〇	〇	〇
		y (Vertical direction)	〇	〇	〇
		z (Medio-lateral direction)		〇	〇
		Rotation about the medio-lateral axis	〇	〇	〇
		Rotation about the anterior–posterior axis		〇	〇
		Rotation about the vertical axis		〇	〇
Right Thigh	Right Hip	Flexion/Extension	〇	〇	〇
		Internal/External Rotation		〇	〇
		Adduction/Abduction		〇	〇
Right Shank	Right Knee	Flexion/Extension	〇	〇	〇
		Internal/External Rotation			〇
		Adduction/Abduction			〇
Right Foot	Right Ankle	Dorsi/Plantar Flexion	〇	〇	〇
		Inversion/Eversion		〇	〇
Left Thigh	Left Hip	Flexion/Extension	〇	〇	〇
		Internal/External Rotation		〇	〇
		Adduction/Abduction		〇	〇
Left Shank	Left Knee	Flexion/Extension	〇	〇	〇
		Internal/External Rotation			〇
		Adduction/Abduction			〇
Left Foot	Left Ankle	Dorsi/Plantar Flexion	〇	〇	〇
		Inversion/Eversion		〇	〇

**Figure 1:**
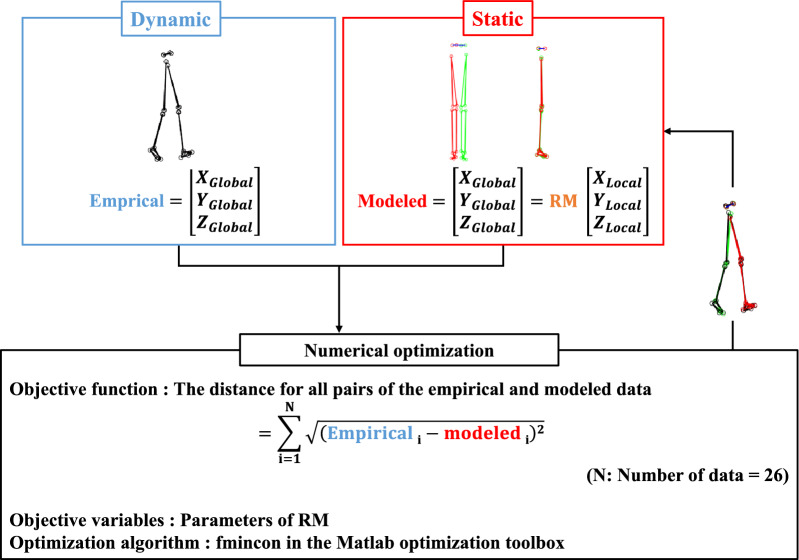
Outline of the nonlinear optimization analysis.

A local coordinate system was defined for each rigid-body segment. The position data of the markers in each local coordinate system were transformed using a simultaneous transformation matrix (STM). A set of parameters for the STM at each frame was determined using a nonlinear optimization algorithm (fmincon in the MATLAB optimization toolbox) to minimize the sum of squares of the Euclidian distance for all pairs of the empirical and modeled data (Fig. 1)^10,11^.

As the AIC tends to select models with a larger number of parameters when the sample size is small, bias-corrected AIC (cAIC) was used^12^. The cAIC values for each model are calculated as follows:

1 $${\text{cAIC value}} = - 2{ } \times { }\left( {MLL} \right) + { }2{ } \times { }DOF + { }\frac{{2DOF\left( {DOF + 1} \right)}}{N - DOF - 1},$$

where *MLL*, *N,* and *DOF* indicate the maximized logarithmic likelihood, the number of reflective markers placed on the lower extremity (i.e., twenty-six in this study), and the DOF for each model, respectively. The MLL is calculated as follows:

2 $$MLL = - \frac{1}{{2\sigma^{2} }}\mathop \sum \limits_{i = 1}^{N} \left( {{\text{PE}}_{i} } \right)^{2} - \frac{N}{2}log2\pi \sigma^{2} ,$$

where PE denotes the distance for a pair of the empirical and modeled data, and ${\sigma }^{2}$ denotes the variance of the PEs. The frame in which the sum of the PEs for M1 reached its maximum during the stride was used to calculate the MLL for each model. As the cAIC value decreases with increasing MLL (i.e., goodness-of-fit of the model) and decreasing number of DOFs (i.e., complexity of the model), the model with the smallest cAIC value is considered the best model, assuming that the goodness-of-fit and simplicity are better balanced than those of the other models.

### Statistical analysis

A two-way repeated-measures ANOVA with two factors—model (9-, 18-, and 22-DOF models) and condition (walking and running)—was used to examine the main and interaction effects on the cAIC and MLL values. When the sphericity assumption was violated, the Greenhouse–Geisser correction was applied. A Bonferroni’s post hoc multiple comparison test was performed if a significant main effect was observed. Indices of effect size (Hedge’s g for pairwise comparisons, partial eta squared ${{\eta }_{p}}^{2}$ for ANOVA) were reported with p-values. A significance level of *p* < 0.05 was used for all comparisons. Statistical analyses were performed using IBM SPSS Statistics for Windows, version 23 (IBM Corporation, Armonk, NY, USA).

## Results

The mean value and standard deviation (SD) of the gait speed (Mean $\pm$ SD) were 1.05 $\pm$ 0.05 m/s and 1.59 $\pm$ 0.16 m/s, for walking and running conditions, respectively.

**Figure 2:**
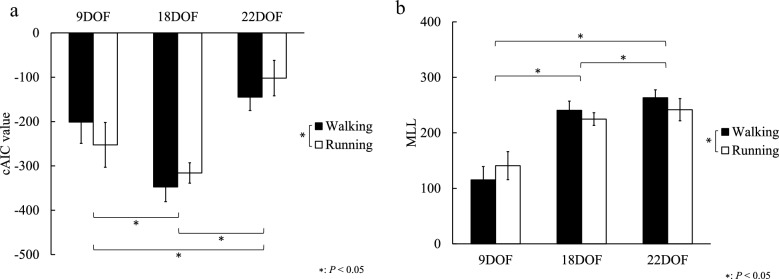
(**a**) cAIC and (**b**) MLL values during walking and running conditions.

A significant interaction between the model and condition was found on the cAIC value (*p* < 0.001, ${{\eta }_{p}}^{2}=0.698$). A significant simple main effect of the model was found for both the walking and running conditions. For the walking condition, pairwise comparisons revealed that the cAIC value of the 18-DOF model was significantly smaller than those of the 9-DOF (*p* < 0.001, $g=3.55$) and 22-DOF (*p* < 0.001, $g = 1.42$) models (Fig. 2a). For the running condition, the cAIC value of the 18-DOF model was also significantly smaller than those of the 9-DOF (*p* = 0.010, $g= 1.62$) and 22-DOF (*p* < 0.001, $g = 3.30$) models (Fig. 2a). A significant simple main effect of the condition was found on the cAIC value for each model. The cAIC value of the 9-DOF model in the walking condition was significantly larger than that in the running condition (*p* = 0.015, $g = 1.04$), whereas those of the 18-DOF (*p* = 0.007, $g = 1.11$) and 22-DOF models (*p* < 0.001, $g = 1.24$) in the walking condition were significantly smaller than those in the running condition (Fig. 2a).

A significant interaction between the model and condition was found on the MLL value (*p* < 0.001, ${{\eta }_{p}}^{2}=0.698$). A significant simple main effect of the model was found for both the walking and running conditions. For the walking condition, pairwise comparisons revealed that the MLL value of the 22-DOF model was significantly larger than those of the 9-DOF (*p* < 0.001, $g= 7.48$) and 18-DOF (*p* < 0.001, $g= 1.47$) models (Fig. 2b). For the running condition, the MLL value of the 22-DOF model was significantly larger than those of the 9-DOF (*p* < 0.001, $g= 4.42$) and 18-DOF (*p* = 0.009, $g= 1.03$) models (Fig. 2b). A significant simple main effect of the condition was also found on the MLL value for each model. For the 9-DOF model, the MLL value in the walking condition was significantly lower than that in the running condition (*p* = 0.015, $g = 1.04$), whereas those of the 18-DOF (*p* = 0.007, $g = 1.11$) and 22-DOF models (*p* < 0.001, $g = 1.24$) in the walking condition were significantly higher than those in the running condition (Fig. 2b).

## Discussion

This study aimed to determine the optimal number of DOFs of the lower extremity model during walking and running. The cAIC values were compared among the models with 9, 18, or 22 DOFs, and the model with the smallest value was considered optimal, indicating a better balance between the goodness-of-fit and generalizability of the model. A significant interaction between the model and condition was observed for the cAIC value. A significant simple main effect of the model and condition was found for the cAIC value. The cAIC value for the 18-DOF model was significantly lower than those of the 9-DOF or 22-DOF models for both conditions. These findings suggest that the 18-DOF model is best suited to represent the lower extremities in terms of the quality of data fitting and its generalizability, although the balance of these two factors in the models varies depending on the type of locomotion.

The significantly larger cAIC values for the 9-DOF model compared with those of the other two models in both walking and running conditions are attributed to its lower goodness of fit, demonstrating the smallest MLL value. As the lower-limb movements were confined to the sagittal plane for the 9-DOF model, the out-of-plane motion appeared to have significantly affected the quality of data fitting. Therefore, the 9-DOF planar model may be oversimplified to represent the lower-limb movements during walking and running because these out-of-plane movements during walking and running cannot be ignored.

On the other hand, the significantly larger cAIC values of the 22-DOF model for both walking and running conditions are attributed to its complexity. Although an increase in the number of DOFs for multi-segmental rigid-body models results in a better quality of data fitting, the generalizability of the model decreases as it becomes more flexible and may therefore become specific for a particular dataset of participants. The 22-DOF model appears to be significantly more complex than the 18-DOF model, thus affecting the generalizability when analyzing the lower-limb movements during walking and running.

The significant interaction between the model and the condition on the cAIC value implies that the quality of the model also varies depending on the type of locomotion. The cAIC value was lower during running than walking for the 9-DOF model, whereas it was lower during walking than running for the 18- and 22-DOF models. This result indicates that the quality of the model considerably decreased when the segmental and joint motions of the lower extremity on the frontal plane were constrained, and its effect was more prominent in walking than running. Since the pelvic tilt and hip adduction movements are major determinants of mediolateral displacement of the whole-body center of mass during walking^13^, the lack of the DOF for these movements might have affected the model's quality of the lower extremity during walking. Therefore, it should be noted that the balance between the model’s quality of data fitting and generalizability differs with different types of locomotion, even though the same model is used for the analysis.

This study had several limitations. For instance, the participants performed walking and running at their preferred speeds. Walking and running speeds may affect the quality of data fitting of the model. It should also be noted that the participants were only healthy male adults of 21–24 years of age. The current findings may not be applicable to other populations because the kinematics of the lower extremities during walking and running varies between different genders and ages and with several types of pathological conditions^14,15^. Further studies are required to determine whether these findings are applicable to other populations and different gait speeds.

In conclusion, the cAIC value of the 18-DOF model was significantly lower than those of the 9- and 22-DOF models for both walking and running. These findings suggest that the 18-DOF model is optimal for analyzing human locomotion in terms of its goodness of fit and generalizability. This finding would help us determine the best model for describing human locomotion when utilizing inverse and forward dynamics analyses.

## Data Availability

The datasets used and/or analyzed during the current study available from the corresponding author on reasonable request.
